# Assessing Different Temporal Scales of Calcium Dynamics in Networks of Beta Cell Populations

**DOI:** 10.3389/fphys.2021.612233

**Published:** 2021-03-23

**Authors:** Jan Zmazek, Maša Skelin Klemen, Rene Markovič, Jurij Dolenšek, Marko Marhl, Andraž Stožer, Marko Gosak

**Affiliations:** ^1^Faculty of Natural Sciences and Mathematics, University of Maribor, Maribor, Slovenia; ^2^Faculty of Medicine, University of Maribor, Maribor, Slovenia; ^3^Faculty of Electrical Engineering and Computer Science, University of Maribor, Maribor, Slovenia; ^4^Faculty of Education, University of Maribor, Maribor, Slovenia

**Keywords:** islets of Langerhans, beta cell network, calcium oscillations, multimodal activity analysis, confocal imaging, functional connectivity, multiplex network

## Abstract

Beta cells within the pancreatic islets of Langerhans respond to stimulation with coherent oscillations of membrane potential and intracellular calcium concentration that presumably drive the pulsatile exocytosis of insulin. Their rhythmic activity is multimodal, resulting from networked feedback interactions of various oscillatory subsystems, such as the glycolytic, mitochondrial, and electrical/calcium components. How these oscillatory modules interact and affect the collective cellular activity, which is a prerequisite for proper hormone release, is incompletely understood. In the present work, we combined advanced confocal Ca^2+^ imaging in fresh mouse pancreas tissue slices with time series analysis and network science approaches to unveil the glucose-dependent characteristics of different oscillatory components on both the intra- and inter-cellular level. Our results reveal an interrelationship between the metabolically driven low-frequency component and the electrically driven high-frequency component, with the latter exhibiting the highest bursting rates around the peaks of the slow component and the lowest around the nadirs. Moreover, the activity, as well as the average synchronicity of the fast component, considerably increased with increasing stimulatory glucose concentration, whereas the stimulation level did not affect any of these parameters in the slow component domain. Remarkably, in both dynamical components, the average correlation decreased similarly with intercellular distance, which implies that intercellular communication affects the synchronicity of both types of oscillations. To explore the intra-islet synchronization patterns in more detail, we constructed functional connectivity maps. The subsequent comparison of network characteristics of different oscillatory components showed more locally clustered and segregated networks of fast oscillatory activity, while the slow oscillations were more global, resulting in several long-range connections and a more cohesive structure. Besides the structural differences, we found a relatively weak relationship between the fast and slow network layer, which suggests that different synchronization mechanisms shape the collective cellular activity in islets, a finding which has to be kept in mind in future studies employing different oscillations for constructing networks.

## Introduction

Rhythmicity is a hallmark of many organs within the human body, a process manifested from molecular reactions to whole body rhythms. An important example is the oscillatory nature of insulin secretion (Lang et al., 1979). Insulin is an anabolic hormone secreted from pancreatic beta cells, mainly postprandially. Since insulin is secreted in the portal vein, the liver is the first organ exposed to it, and up to 80% of secreted insulin is cleared by the liver by the receptor-mediated process during the first liver passage (Eaton et al., 1983). The amplitude of insulin release directly defines hepatic insulin clearance as well as consecutive systemic insulin amount (Meier et al., 2005). The oscillatory pattern of insulin delivery to the target tissues is essential for insulin action, ensuring a higher level of sensitivity of target tissues compared with the same amount of insulin administered at a constant dose (Matthews et al., 1983b). In the liver, pulsatile insulin delivery suppresses hepatic glucose production more effectively (Matveyenko et al., 2012) and prevents insulin receptor desensitization (Li and Goldbeter, 1992). Fluctuations in hepatic glucose production determine oscillations in the plasma glucose concentration, representing a possible feedback mechanism for pancreatic insulin secretion (Goodner et al., 1982; Pedersen et al., 2005). The changes in the normal pattern of plasma insulin oscillations are an early marker of insulin resistance and diabetes mellitus and can be found in diabetic animal models, such as *ob/ob* mice (Ravier et al., 2002) and ZDF rats (Sturis et al., 1994), as well as in diabetic patients (Lang et al., 1981; Polonsky et al., 1988) and even their relatives (O’rahilly et al., 1988). Besides disrupted pulsatility of insulin release, the hepatic extraction of insulin is impaired in diabetic patients (Sando et al., 1980; Bonora et al., 1983).

Insulin concentration *in vivo* oscillates with a period of 5–15 min (Matthews et al., 1983a; Porksen et al., 1995; Song et al., 2000) and elevated plasma glucose increases the amplitude but not the frequency of plasma insulin oscillations (Matthews et al., 1983a; Juhl et al., 2000). It has been confirmed that oscillations of plasma insulin can be due to an intrinsically pulsatile release of insulin from the pancreas (Stagner et al., 1980). The oscillatory nature of insulin release has also been observed in isolated islets, further suggesting that oscillatory insulin secretion does not rely on external or intrapancreatic neural stimulation, but it is an intrinsic property of pancreatic islets, although several external factors may modulate it *in vivo* (Dean and Matthews, 1970; P. Gilon et al., 1993). However, there are still some open questions regarding the regulation and synchronization of insulin release from individual beta cells within an islet and between different islets that enable the appearance of pulsatile plasma insulin levels with a period of 5–15 min. Besides these oscillations, slower ultradian rhythms with a period of about 2 h (Simon et al., 1987) and circadian rhythms of insulin secretion, have also been observed (Peschke and Peschke, 1998).

The stimulus-secretion coupling in pancreatic beta cells involves the entry of glucose into the cell and glucose metabolism, resulting in increased ATP, which in turn decreases the open probability of ATP-dependent potassium (K_*ATP*_) channels (Nilsson et al., 1996). This brings about membrane depolarization, the opening of voltage-dependent Ca^2+^ channels, and increased cytosolic Ca^2+^ concentration, which triggers the beta cell secretory machinery and insulin secretion. In addition to these so-called triggering pathways, additional metabolic and neurohormonal pathways exist (Henquin, 2011; Skelin Klemen et al., 2017). Individual beta cells respond to increased glucose concentration with oscillations in membrane potential, Ca^2+^, and insulin secretion. Mouse beta cells in isolated islets, in pancreas tissue slices, and *in vivo* oscillate at three different temporal scales when exposed to stimulatory glucose concentration above 6 mM glucose (Santos et al., 1991; Gilon and Henquin, 1992; Bergsten et al., 1994; Dolenšek et al., 2013; Stožer et al., 2013; Salem et al., 2019; Jacob et al., 2020). The slowest Ca^2+^ oscillations with a frequency of 0.06–0.2 min^–1^ and duration of 5–15 min lie in a range similar to the plasma insulin oscillations and are thought to underlie the pulsatility in plasma insulin. These slow oscillations most probably reflect metabolic activity and drive the oscillatory ATP production, which in turn affects the intermittent activity of K_*ATP*_-channels (Nilsson et al., 1996; Tornheim, 1997). In pancreatic beta cells, like in many other living cells, the phosphofructokinase-catalyzed step is one of the candidates responsible for the oscillatory nature of the metabolic activity (Westermark and Lansner, 2003) and has been found crucial for normal insulin secretion (Ristow et al., 1999). Superimposed on the slow oscillations are the so-called fast Ca^2+^ oscillations with a frequency of about 5 min^–1^ and a duration of about 2–15 s. It is currently believed that these oscillations result from Ca^2+^ feedback on ion channels, and therefore reflect the bursting pattern of electrical activity. The frequency and the duration of these oscillations are glucose-dependent (Meissner, 1976; Santos et al., 1991; Nunemaker et al., 2006; Scarl et al., 2019; Dolenšek et al., 2020) and are considered essential for setting the amplitude of the slow plasma insulin oscillations (Bergsten, 2002; Hellman, 2009; Satin et al., 2015). Both slow and fast oscillations are well synchronized between different beta cells of the same islet (Satin et al., 2015; Skelin Klemen et al., 2017; Bertram et al., 2018). Finally, it should be noted that there exist even faster Ca^2+^ oscillations with a duration of around 100 ms, which are superimposed on the fast oscillations or bursts and are called spikes. They correspond to individual action potentials, observed during a burst of membrane potential depolarization.

To ensure the pulsatile profile of plasma insulin, both inter- and intra-islet synchronization seem to be essential. How different islets within the pancreas are coordinated to produce pulsatile plasma insulin is still not completely understood. Since plasma glucose fluctuates with a similar period as plasma insulin (Lang et al., 1979), the glucose feedback to pancreatic islets could account for the synchronization of the islets (Westerlund and Bergsten, 2001; Gilon et al., 2002; Pedersen et al., 2005). Besides classical feedback mechanisms, neural mechanisms with parasympathetic and sympathetic neurons exhibiting the opposite effect on islet function (Ahrén, 2000) and signals from other non-pancreatic tissues, like the intestine (Drucker, 2007), liver (Imai et al., 2008), fat tissue (Morioka et al., 2007), bones (Lee et al., 2007), and others, seem important for normal islet function (Eberhard and Lammert, 2009). On the other hand, synchronization between individual beta cells within a single islet is believed to be achieved *via* gap-junctional coupling through Connexin36 and through additional means of intercellular communication (Meda et al., 1979; Moreno et al., 2005; Eberhard and Lammert, 2009; Benninger et al., 2011; Almaça et al., 2020). This coupling enables neighboring beta cells to communicate and, in part, synchronize their dynamics. The diffusion of intermediate products of glycolysis, in particular glucose-6-phosphate, is probably responsible for the coupling of slow oscillations (Tsaneva-Atanasova et al., 2006), while electrical depolarization with a space constant in the order of a few beta cell diameters accounts for the alignment of fast oscillations and explains the experimentally observed Ca^2+^ waves (Meissner, 1976; Meissner and Preissler, 1979; Eddlestone et al., 1984; Santos et al., 1991; Aslanidi et al., 2001; Benninger et al., 2008; Zhang et al., 2008; Skelin Klemen et al., 2017; Šterk et al., 2021). Furthermore, it was proposed that the electrical coupling increases with glucose concentrations (Eddlestone et al., 1984).

Investigating the collective activity of beta cell populations is gaining attention, primarily because of the increasing amount of data showing that the pathogenesis of diabetes comprises disruptions of regulated collective cellular activity and the consequent disturbance in insulin secretion (Head et al., 2012; Hodson et al., 2013; Skelin Klemen et al., 2017; Westacott et al., 2017a; Adams et al., 2020; Akalestou et al., 2020). However, the pancreatic islets are characterized by multiple facets of complexity in the cytoarchitecture and cellular dynamics, as well as with the presence of heterogeneity and biological variability, which makes the overall function of these highly interconnected structures difficult to understand. Noteworthy, in the last few years, combining the complex networks theory with advanced imaging techniques has proven to be an advantageous tool for quantifying multicellular dynamics in these micro-organs (Hodson et al., 2013; Stožer et al., 2013; Johnston et al., 2016; Gosak et al., 2018; Salem et al., 2019). By these means, functional networks constructed on the basis of statistical similarity between simultaneously measured signals of multiple cells are used to embody intercellular communication patterns. The methodology was not only found useful for demonstrating that beta cell networks share many similarities with several other biological networks, such as small-worldness, modularity, and a heterogeneous degree distribution (Stožer et al., 2013; Johnston et al., 2016; Gosak et al., 2018), but also that there are important relations between beta cell metabolic activity and the orchestration of collective islet behavior (Gosak et al., 2015; Johnston et al., 2016). Moreover, it turned out that beta cell networks are rather segregated, which is most probably linked to cellular variability and the existence of sub-populations (Markovič et al., 2015; Dwulet et al., 2019; Dolenšek et al., 2020; Nasteska et al., 2020). The beta cell connectivity architectures were also found to be very heterogeneous with a small fraction of very well connected cells, i.e., hub cells, which are believed to substantially affect the collective cellular activity (Johnston et al., 2016; Lei et al., 2018; Loppini and Chiodo, 2019; Salem et al., 2019; Nasteska et al., 2020), even though the precise mechanisms are still incompletely understood (Satin et al., 2020). Therefore, how various intercellular coupling mechanisms and the interplay between electrical and metabolic activity in populations of heterogeneous cells shape the complex spatio-temporal dynamics in islets and how these functions are impaired in diabetes is a matter of ongoing research. One of the main limitations in the field of complex network approaches to understanding beta cell synchronization is that different groups employ different types of Ca^2+^ oscillations as the basis for constructing functional networks.

In the present study, we, therefore, aim to further explore the multimodal nature of oscillatory activity in pancreatic beta cells that is governed by interactions of various physiological regulatory systems. We distinguish between the metabolically driven low-frequency component of Ca^2+^ oscillations (order of minutes) and the high-frequency component, which is governed by the membrane electrical activity (order of seconds). We focus particularly on the relationship between both oscillatory components and to what extent their collective rhythmicity is coordinated on the multicellular level. For this purpose, we combine time series analysis with network-theoretical approaches to examine glucose-stimulated oscillatory Ca^2+^ dynamics measured in beta cells from acute mouse pancreas tissue slices.

## Materials and Methods

### Ethics Statement

The study was carried out in strict accordance with all national and European recommendations related to work with experimental animals, and all efforts were made to minimize the suffering of animals. The protocol was approved by the Administration of the Republic of Slovenia for Food Safety, Veterinary Sector and Plant Protection (permit number: 34401-35-2018/2).

### Tissue Slice Preparation

Pancreas tissue slices were prepared from adult NMRI male mice kept in individually ventilated cages (Allentown, PA, United States) on a 12 light/12 dark cycle, as described previously (Speier and Rupnik, 2003; Stožer et al., 2013). In brief, after sacrificing the animals by a high concentration of CO_2_, the abdomen was exposed *via* laparotomy and low-melting-point 1.9% agarose (Lonza Rockland Inc., Rockland, ME, United States) in extracellular solution (ECS, consisting of (in mM) 125 NaCl, 26 NaHCO_3_, 6 glucose, 6 lactic acid, 3 myo-inositol, 2.5 KCl, 2 Na-pyruvate, 2 CaCl_2_, 1.25 NaH_2_PO_4_, 1 MgCl_2_, 0.5 ascorbic acid continuously bubbled with a gas mixture containing 95 % O_2_ and 5 % CO_2_ at barometric pressure to ensure oxygenation and a pH of 7.4) at 40°C was retrogradely injected into the pancreatic ductal tree *via* the proximal common bile duct clamped at the papilla of Vater. Subsequently, following immediate cooling with ice-cold ECS and extraction, small blocks of tissue (0.1–0.2 cm^3^ in size) were cut and embedded in agarose at 40°C. The tissue was cut at 0.05 mm s^–1^ and 70 Hz into 140 μm-thick slices (VT 1000 S vibratome, Leica, Nussloch, Germany), and the obtained slices collected in HEPES-buffered saline at room temperature (HBS, consisting of (in mM) 150 NaCl, 10 HEPES, 6 glucose, 5 KCl, 2 CaCl_2_, 1 MgCl_2_; titrated to pH = 7.4 using 1 M NaOH) until incubation in the dye-loading solution. All chemicals were obtained from Sigma-Aldrich (St. Louis, MO, United States) unless indicated.

### Dye Loading and Ca^2+^ Imaging

Slices were incubated in the dye-loading solution [6.87 μM Calbryte 520AM (Calbryte, AAT Bioquest, CA, United States), 0.03% Pluronic F-127 (w/v), and 0.12% dimethylsulfoxide (v/v) dissolved in HBS] at RT for 50 min. Following the staining protocol, the slices were transferred into HBS containing 6 mM glucose and stored for up to 8 h until Ca^2+^ imaging. For Ca^2+^ imaging, individual tissue slices were transferred to the perfusion system delivering carbogenated ECS with varying glucose concentrations, according to the stimulation protocol, and kept at 37°C. The protocol consisted of initial exposure to the non-stimulatory 6 mM glucose, followed by either 8 or 12 mM glucose for 45 min, and washout with 6 mM glucose. The Ca^2+^ imaging was performed on a Leica TCS SP5 AOBS Tandem II upright confocal system (20x HCX APO L water immersion objective, NA 1.0) and a Leica TCS SP5 DMI6000 CS inverted confocal system (20X HC PL APO water/oil immersion objective, NA 0.7). The acquisition was set to 10 Hz at 512 × 512 pixels to make the precise quantification of Ca^2+^ oscillations feasible. The dye was excited by argon 488 nm laser line and emitted fluorescence was detected by Leica HyD hybrid detector in the range of 500–700 nm (all from Leica Microsystems, Germany), as described previously (Stožer et al., 2013). Additionally, a higher resolution (1,024 × 1,024 pixels) image was acquired. Beta cells identification was done by selecting regions of interest (ROIs) off-line using microscope software or third-party software. ROIs were selected based on cell morphology using a higher resolution image or alternatively, based on maximal projection image from time series and cell activity observed by replaying the time-lapse videos. Time-series data were corrected for photobleaching, employing a combination of linear and single exponential fit, and signals were expressed as (*F*–*F*_0_)/*F*_0_ ratios, where *F*_0_ is the initial fluorescence intensity, and *F* is the fluorescence signal recorded at an individual time point during the experiment.

### Processing of Recorded Ca^2+^ Traces

The recorded time series of Ca^2+^ signals were first corrected for photobleaching of the dye employing a combination of linear and single exponential fit as described previously (Stožer et al., 2013). A Butterworth filter of the 5th order was then used to extract the fast and slow dynamical component from the recorded signals. To attain the low-frequency, i.e., slow, component, we applied the band-pass filter with 1×10^−3^ and 5×10^−3^ Hz for the lower and upper cutoff frequency, respectively. For the high-frequency, i.e., fast, component, we used 4×10^−2^ and 4×10^−1^ Hz for the lower and upper cutoff frequency, respectively.

**FIGURE 1:**
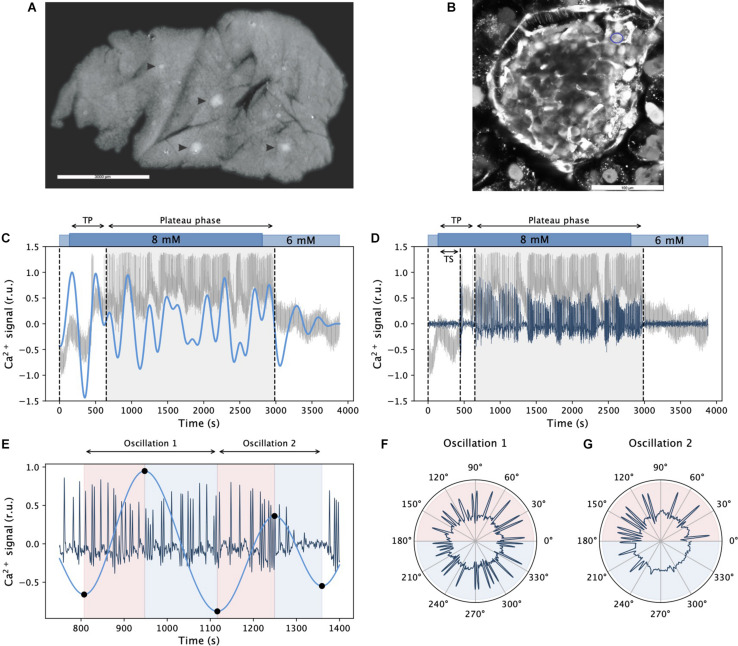
Inferring dynamical components of beta cell calcium activity. Panel **(A)** shows an acute pancreas tissue slice under the stereomicroscope with islets of Langerhans indicated by arrowheads. In panel **(B)**, a representative confocal image with well stained cells in the islet of Langerhans using calcium dye Calbryte 520AM is presented. The blue circle indicates a beta cell. In panels **(C,D)**, a raw recorded trace from an exemplary cell is shown in gray after stimulation with 8 mM glucose. Blue lines in panels **(C,D)** signify the extracted slow and fast component signals, respectively. The gray shaded areas denote the plateau phase of sustained activity, TS and TP specify the activation delay and the onset of sustained activity, respectively. Panel **(E)** visualizes the ascending and descending phases of two succeeding slow component oscillations and the corresponding activity of fast oscillations. Black dots on the curve denote local extremes, and the colored background indicates the corresponding ascending and descending phases. In panels **(F,G)**, polar plots show the activity of fast oscillations with respect to the phase of the slow component oscillations featured in panel **(E)**.

For further analyses, we discretized both dynamical components. The fast component was binarized so that the values from the onset to the end of individual oscillations were 1, and values between the oscillations were 0. The binarized signals were then used to characterize the fast oscillatory activity, i.e., to calculate the average frequency, the average duration of oscillations, and the relative active time. The latter defines the fraction of time that the cells spend in an active state with increased intracellular Ca^2+^. Moreover, each oscillation of the slow component, i.e., the interval between two local maxima, was discretized to 12 segments, representing the phase intervals of the pseudo-sinusoidal wave function. More specifically, the time of the *j*-th local minimum and *j*-th local maximum of the *i*-th cell is denoted by $t_{i,j}^{\text{min}}$ and $t_{i,j}^{\text{max}}$, respectively. We divided the ascending part of the slow component $\left[t_{i,j}^{\text{min}},t_{i,j}^{\text{max}}\right]$ into six equidistant intervals and assigned values 1,2,…,6 (corresponding to the phase intervals $\left[0,\frac{\pi }{6}\right],\left[\frac{\pi }{6},\frac{\pi }{3}\right],\ldots ,\left[\frac{5\pi }{6},\pi \right]$. Similarly, we divided the descending part of the slow component $\left[t_{i,j}^{\text{max}},t_{i,j+1}^{\text{min}}\right]$ into 6 equidistant intervals and assigned values 7,8,…,12 (corresponding to the phase intervals $\left[\pi ,\frac{7\pi }{6}\right],\left[\frac{7\pi }{6},\frac{4\pi }{3}\right]\ldots \left[\frac{11\pi }{6},2\pi \right]$). See Figures 1C–E for further insight.

### Functional Network Analysis

Based on the extracted fast and slow dynamics of individual cells, we construct the corresponding fast and slow functional network layers. Nodes represent individual beta cells, and their positions correspond to physical locations of cells in tissue slices. Edges between node pairs are created on the basis of the temporal similarity of Ca^2+^ dynamics, given with the correlation coefficient between the *i*-th and *j*-th cell, *R*_*i,j*_, computed as:

$$R_{i,j}=\frac{\sum_{t=0}^{T}\left[f_{i}\left(t\right)-\bar{f_{i}}\right]\left[f_{j}\left(t\right)-\bar{f_{j}}\right]}{\text{std}\left(f_{i}\left(t\right)\right)\text{std}\left(f_{j}\left(t\right)\right)},$$

where *f*_*i*_(*t*) and *f*_*j*_(*t*) represent the slow or fast traces of the *i*-th and *j*-th cell. By computing *R*_*i,j*_ among all node pairs, we create the correlation matrix, *R*, with the *ij*-th element being the correlation coefficient *R*_*i,j*_. To enable a direct comparison between different networks, we used variable thresholds to extract the binary adjacency matrix, so that the average node degree in each network was *k=8*. Conventional tools from the complex network theory were then used to quantify functional beta cell networks (Boccaletti et al., 2006), as described previously (Gosak et al., 2018). In brief, the relative degree distribution was calculated to explore the connectivity of cells in different network layers. For the evaluation of the network’s functional segregation, we computed the average clustering coefficient and modularity, which reflect the level of clique-like structures within interconnected cell assemblies and the extent of division into smaller subpopulations, respectively. To characterize the level of functional integration, we computed the relative largest component, which quantifies the fraction of cells in the islet that are either directly or indirectly connected. In addition, we calculated the average physical length of functional connections.

### Statistical Analysis

Statistical analyses were performed using the statistics package in SigmaPlot 11 (Systat, Software Inc., IL, United States). We compared groups by using the *t*-test or the Mann–Whitney test (for non-normally distributed data). All significances are expressed as exact values and the number of islets included in analyses indicated accordingly. All significances are expressed as exact *p* values (*p*) and the number of islets included in analyses is indicated accordingly. We estimated effect sizes by calculating the values of Cohen’s *d* (*d*) by dividing the difference in sample means by the pooled standard deviation, according to the original definition (Cohen, 2013). Our judgements about effect sizes are in accordance with a recent classification (Sawilowsky, 2009).

## Results

We studied the effect of stimulation with two glucose concentrations: a physiological concentration that is commonly observed *in vivo*, i.e., 8 mM, and a supraphysiological concentration, i.e., 12 mM. First, we focused on the temporal aspect of the glucose-evoked oscillatory Ca^2+^ activity measuring the classical physiological measures, whereas in the second part of our analyses, we examined the synchronicity and collective activity of beta cell populations utilizing correlation analysis and network-based approaches. Particular emphasis was devoted to the interrelationship between the slow and the fast oscillatory component in both approaches.

### Assessing the Multimodal Oscillatory Intracellular Ca^2+^ Activity in Pancreatic Beta Cells

In acute mouse pancreas tissue slice, islets of Langerhans are recognized under the stereomicroscope as white spots (Figure 1A) and therefore easily distinguished from the surrounding exocrine tissue. In an individual tissue slice up to five or six islets could be found, but only one of these islets per slice was used for calcium imaging. This islet was selected based on the size, successful loading with calcium dye and preserved architecture of the islet. A representative confocal image of an islet used for calcium imaging is shown on Figure 1B. Pancreatic beta cell Ca^2+^ response to glucose stimulation was recorded by means of multicellular confocal imaging in acute tissue slices as described in Materials and methods. The cells responded to stimulation with a delay in the onset of Ca^2+^ activity (TS), and they reached a state of sustained activity after a slightly longer time interval (TP, Figures 1C,D). The latter is termed the plateau phase and is characterized by repetitive well-aligned fast oscillations lasting a few seconds (MacDonald and Rorsman, 2006; Stožer et al., 2013, 2019). Most importantly, as it can be inferred from the recorded Ca^2+^ traces (gray lines in Figures 1C,D), this fast oscillatory activity is superimposed on a low-frequency oscillatory component. Using proper band-pass filters, we could extract individual dynamical components from the raw signals (see blue lines in Figures 1C,D for the slow and the fast component, respectively). At first glance, it can be observed that there was an order of magnitude difference in the frequency of both components and that the low-frequency component correlated with the behavior of the high-frequency oscillations, which will be addressed in more detail in continuation. To explore the relationship between both oscillatory components, we defined individual phases of slow oscillations, as illustrated in Figure 1E. This way, the activity of fast oscillations could be studied in the context of the slow component phases, as presented in Figure 1F.

**FIGURE 2:**
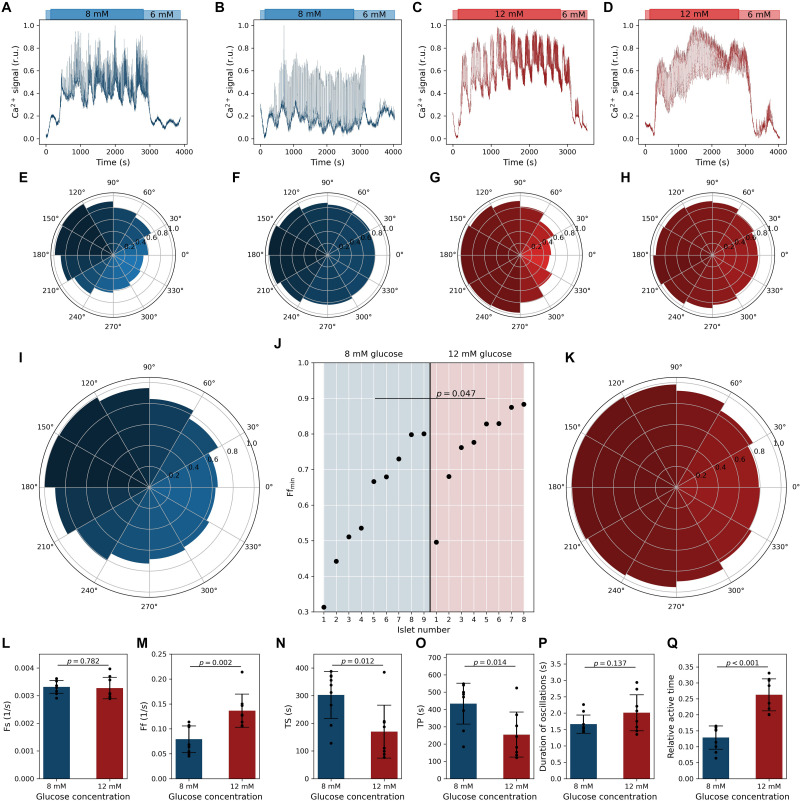
Quantifying oscillatory intracellular Ca^2+^ activity in pancreatic beta cells. Dynamics of two different oscillatory phenotypes of pancreatic beta cells stimulated with 8 mM glucose **(A,B)** and 12 mM glucose **(C,D)**. Blue and red lines in panels **(A–D)** show average calcium signals of all beta cells in a given islet (before filtering). Panels **(E–H)** feature the corresponding relative phase-dependent activity of fast oscillations, i.e., relative density of fast oscillations at different phases of the slow oscillatory component. Islets presented in panels **(A,C)** exhibit well-pronounced slow oscillations, and the frequency of fast oscillations depends profoundly on the phase of the slow component. In contrast, the islet in presented panels **(B,D)** exhibits rather weakly pronounced slow oscillations and a very subtle slow-phase-dependency of fast oscillations. Panels **(I,K)** show the relative phase-dependent activity of fast oscillations obtained from pooled data of all beta cells and all islets for 8 and 12 mM glucose concentrations, respectively (927 traces from 9 islets for 8 mM glc and 743 traces and 8 islets for 12 mM glc), irrespective of their response phenotype. In panel **(J)**, the lowest value of slow-phase-dependent relative density of fast oscillations, Ff_*min*_, is shown separately for each islet and both glucose concentrations. Cohen’s *d* value is 1.05. Islets 2 and 8 for 8 mM and 1 and 4 for 12 mM correspond to data from panels **(E–H)**. Panels **(L–R)** feature the calcium signaling parameters: frequency of slow oscillations Fs **(L)**, frequency of fast oscillations Fs **(M)**, time required for the cells to respond to stimulation TS **(N)**, time required for the cells to reach the phase of sustained activity, i.e., the plateau phase, TP **(O)**, duration of individual oscillations **(P)**, and the average active time **(R)**. Cohen’s *d* values are 0.14 **(L)**, 1.80 **(M)**, 1.38 **(N)**, 1.36 **(O)**, 0.76 **(P)**, and 2.89 **(Q)**. Black dots denote average values in individual islets, error bars reflect the standard deviation, and the column height displays the average value over all islets.

Ca^2+^ activity of four exemplary cells from different islets is presented in Figures 2A,B for stimulation with 8 mM glucose and in Figures 2C,D for stimulation with 12 mM glucose. In panels below (Figures 2E–H), the corresponding polar density plots displaying the average relative density of fast oscillations as a function of the phase of the slow component are shown. Each plot includes the behavior of all cells and all slow oscillations in the given islet. It can be noticed that in both glucose concentrations, the relationship between the fast and slow oscillatory part can be either well-defined (see Figures 2A,E,C,G) or pronounced only weakly (see Figures 2B,F,D,H). However, irrespective of how apparent the correlation between both oscillatory components was, a very similar phase-dependency of the fast oscillations was attained. Namely, in all four cases, the highest density of fast oscillations was detected around the maxima and the lowest around the minima of slow oscillations. To provide a general insight into this behavior and investigate how it depends on the glucose concentration, we plot in Figures 2I,K, the average phase relationship pooled from all islets for a given glucose concentration. Evidently, a rather strong phase-dependency was observed in 8 mM glucose, whereas the correlation between the fast Ca^2+^ activity and the phase of the slow oscillations was, on average, only weakly pronounced in 12 mM glucose. To elaborate on this issue further, we show in Figure 2J the minimal value in the phase plots for each islet. This number reflects to what extent the frequency of fast oscillations is modulated by the slow oscillatory component. It can be observed that in 8 mm glucose, there was a very broad spectrum of oscillatory phenotypes, whereas, under 12 mM glucose in the majority of the islets, the relationship between the fast and slow oscillations was rather weak, as reflected by significantly higher values of the Ff_*min*_ parameter in 12 mM glucose and by a large effect size (*p* = 0.047, *d* = 1.05).

Next, we quantified the Ca^2+^ signaling parameters, separately for each glucose concentration. In both glucose concentrations the frequency of slow oscillations was around 0.2 min^–1^ (Figure 2L) and was not affected by the stimulation level (the difference was insignificant and the effect size very small, *p* = 0.782, *d* = 0.14). In contrast, the frequency of fast oscillations depended significantly and with a very large effect size (*p* = 0.002, *d* = 1.80) on the stimulation level and was, on average, 4.8 and 8.4 min^–1^ in 8 and 12 mM glucose, respectively (Figure 2M). The durations of individual oscillations tended to be higher under 12 mM glucose, but due to rather high levels of variability, the difference did not reach statistical significance despite a medium effect size (Figure 2P). However, the relative active time, a metric being affected by both frequency and duration, was almost twofold higher under higher stimulatory conditions (Figure 2Q). This difference was significant and characterized by a huge effect size (*p* < 0.001, *d* = 2.89). Apparently, only the activity of the fast oscillatory component is modulated by stimulatory glucose levels. Finally, we characterized the beta cell responses to stimulation by calculating the average time lag until the cells in a given islet responded to stimulation, TS, and the average time required for the cells to reach the phase of sustained activity, i.e., the plateau phase, TP. Both of these parameters were significantly higher under lower stimulation levels and the effect sizes were very large (*p* = 0.012, *d* = 1.38; *p* = 0.014, and *d* = 1.36). In 8 mM, values of TS and TP were approximately 5 and 8 min, whereas in 12 mM glucose, they shortened on average almost twofold (Figures 2N,O). These results are in good agreement with our previous reports (Dolenšek et al., 2020; Podobnik et al., 2020).

It should be noted that the beta cell activity is well synchronized between different cells in the same islet, particularly in the phase of sustained activity. We have therefore used the islet averages to statistically evaluate the differences in cellular signaling parameters. However, to gain a more detailed insight and to additionally assess the intra- and inter-islet variability, we present in Supplementary Figures 1–7 separate data for all islets and the results that are based on pooling data from individual cells. The results reveal that in the domain of fast oscillatory activity the intra-islet variability is clearly lower than inter-islet variability for both glucose concentrations. For the frequency of slow oscillations, for the relationship between the fast and the slow component, and for the time required for the cells to reach the plateau phase, no such obvious conclusions can be drawn. Most importantly, irrespective of the signaling parameter, single-cell-based analyses corroborate the main findings that are based on islet averages, but due to very large sample sizes with a much higher statistical significance.

### Synchronicity and Network Analysis of Multiple Oscillatory Rhythms in Beta Cell Collectives

**FIGURE 3:**
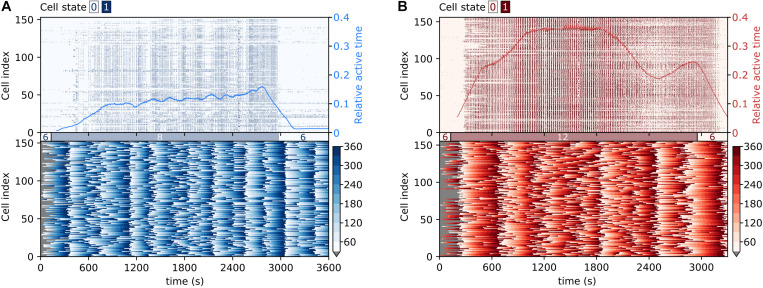
Visualization of collective fast and slow beta cell activity. Raster plots of binarized fast Ca^2+^ oscillations (upper row) and the corresponding color-coded phases of the slow oscillatory activity (lower row) in typical islets stimulated with 8 mM **(A)** and 12 mM **(B)** glucose. The blue and red lines superimposed on the raster plots denote the temporal evolution of the average relative active time, as specified by the scale on the right-hand side of the graph.

To capture the collective temporal activity patterns of beta cell populations, we show in Figure 3 raster plots of binarized fast Ca^2+^ activity and color-coded values of the phases of the slow oscillatory component, for two exemplary islets stimulated with different glucose concentrations. In the domain of fast oscillations, following either of the stimuli, beta cells exhibited a biphasic response (Pedersen et al., 2019; Stožer et al., 2019; Jaffredo et al., 2021). In the first activation phase, the cells were progressively recruited, and Ca^2+^ waves of different sizes were noticed. In the subsequent plateau phase, the islet activity was characterized by dominating global Ca^2+^ waves and rather regular oscillations. Moreover, under physiological stimulation levels, the transition period to the plateau phase was considerably shorter than under supraphysiological stimulation (see also parameter TP in Figure 2O). The collective intercellular activity is also visualized in Supplementary Videos 1, 2, showing animations of binarized spatiotemporal Ca^2+^ dynamics in representative islets. Evidently, the fast oscillatory activity was well-coordinated and spread across the islets in the form of rather well organized and directed Ca^2+^ waves. In the domain of slow oscillations, synchronized spatiotemporal dynamics was observed as well (see lower panels in Figure 3 and Supplementary Videos 3, 4), which, however, is qualitatively different from the fast Ca^2+^ waves. In general, the slow oscillatory events were more global and encompassed the whole islet, but the oscillations were phase-shifted. Most importantly, these shifts changed with time, and there seemed to be a tendency of nearby cells being less phase-shifted than remote ones, although distant cells were found to be in the same phases as well. In continuation, we explore these complex and coherent spatiotemporal patterns in more detail.

**FIGURE 4:**
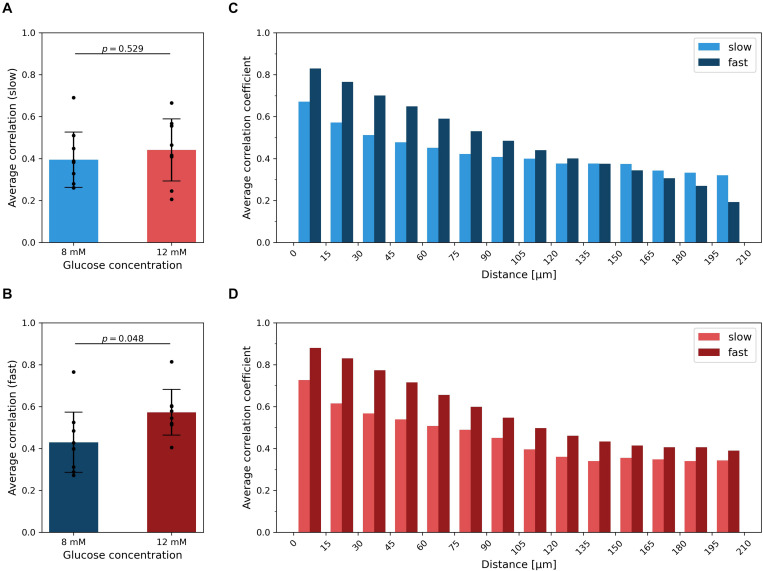
Intercellular synchronicity of slow and fast oscillatory beta cell activity. The average correlation coefficient between the slow **(A)** and fast **(B)** oscillatory components for both glucose concentrations. Black dots denote average values of individual islets, error bars reflect the standard deviation, and the column height displays the average over all islets in the given group. Cohen’s *d* values are 0.31 and 1.05 for average correlation of slow and fast component, respectively. In panels **(C,D)**, the average correlation as a function of the Euclidean distance between cell pairs is shown for 8 mM **(C)** and 12 mM **(D)** glucose, separately for each dynamical component. The heights of the columns represent the average over all cell pairs in all islets belonging to a certain spatial interval.

To characterize the level of synchrony in the slow and fast temporal scales of Ca^2+^ dynamics in islets, we computed the average correlation coefficient for all possible pairs of cells within individual islets, separately for each oscillatory component and for both glucose concentrations. On the scale of slow oscillations, the difference in average synchronicity at different stimulation levels was not significant and the effect size was small (*p* = 0.529, *d* = 0.31) (Figure 4A). On the contrary, higher glucose concentrations evoked more synchronized responses of the fast component (Figure 4B), which corroborates our previous findings (Markovič et al., 2015; Gosak et al., 2018; Dolenšek et al., 2020). The difference was statistically significant and the effect size large (*p* = 0.048, *d* = 1.05). On average, the correlation of the fast oscillatory activity was higher when compared to the slow component. For both dynamical components, the average correlation between cell pairs is a monotonically decreasing function of the intercellular distance, irrespective of the stimulatory glucose concentration (Figures 4C,D). In other words, the correlation in Ca^2+^ activity between nearby cells was roughly twice as high as between remote ones, for both the fast and the slow component. However, for the fast component, this result is expected, because well-defined propagating Ca^2+^ waves serve as the main synchronizing mechanism (Aslanidi et al., 2001; Benninger et al., 2008; Santos et al., 1991; Šterk et al., 2021). For that reason, the average correlation also decreases slower with increasing distance under 12 mM than under 8 mM glucose, since supraphysiological levels of stimulation evoke mainly global waves, which give rise to higher correlations also at higher intercellular distances. In contrast, under physiological glucose levels, there is also a certain fraction of localized Ca^2+^ waves, which do not facilitate global synchronicity (Stožer et al., 2019). Notably, a similar trend was observed for the slow oscillatory component as well, except that the average level of intercellular synchrony was lower. This result does not only corroborate previous observations of slow activity being often coordinated among nearby cells (see Figure 3 and Supplementary Videos 3, 4), but also implies that intercellular communication has an important role by orchestrating the collective activity of the slow oscillatory behavior as well.

**FIGURE 5:**
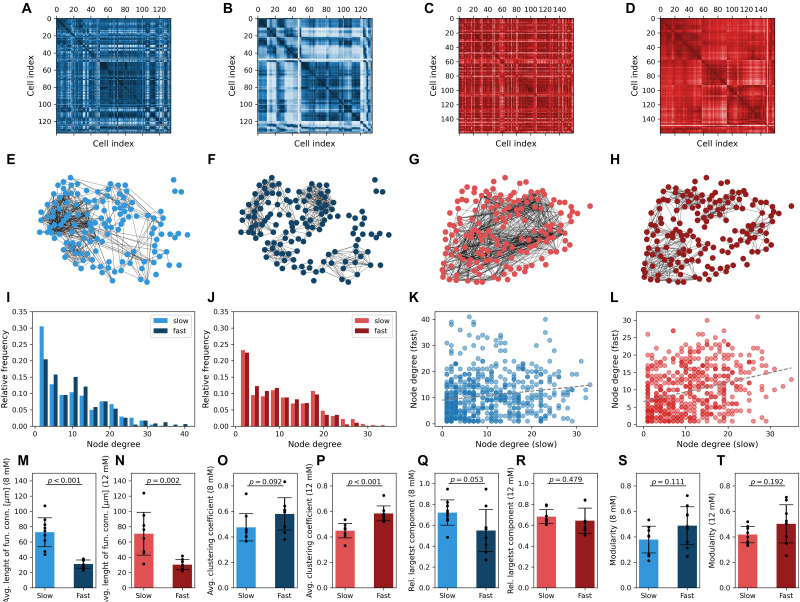
Multiplex network representation and analysis of functional beta cell connectivity maps. Matrix plots of pairwise correlation coefficients in a typical islet stimulated with 8 mM **(A,B)** or 12 mM **(C,D)** glucose, separately for the slow **(A,C)** and fast **(B,D)** oscillatory components. The corresponding functional beta cell network representation is shown in panels **(E–H)** for islets stimulated with 8 mM **(E,F)** and 12 mM **(G,H)** glucose. Nodes signify positions of beta cells within an islet, and connections stand for functional associations between the slow **(E,G)** and fast **(F,H)** oscillatory components. Panels **(I,J)** display the degree distributions for all islets stimulated with 8 mM **(I)** and 12 mM **(J)** glucose, separately for the slow and fast components. Note that the average degree of functional networks in all islets was set to *k* = 8. The correlation between node degrees in the fast- and slow component derived networks is featured in panels **(K,L)** for 8 and 12 mM glucose stimulation, respectively. Only nodes with *k* >  0 were considered. The gray dotted lines represent a linear fit (*R*^2^ was 0.15 for low and 0.31 for high stimulatory conditions, *p* <  0.001). Panels **(M–T)** feature the average pooled data of network parameters for all islets under the given stimulatory glucose concentration, separately for both oscillatory components: physical length of functional connections **(M,N)**, average clustering coefficient **(O,P)**, global efficiency **(R,Q)**, modularity **(S,T)**. Cohen’s *d* values: 2.82 **(M)**, 1.86 **(N)**, 0.85 **(O)**, 2.20 **(P)**, 0.98 **(Q)**, 0.36 **(R)**, 0.79 **(S)**, and 0.70 **(T)**.

To further characterize the collective beta cell activity of both dynamical components, we constructed functional connectivity profiles for both glucose concentrations. Both types of Ca^2+^ traces from all cells were statistically compared in a pairwise manner to build correlation matrices (Figures 5A–D). Functional networks are shown in Figures 5E–H and were obtained by adjusting the connectivity thresholds, so the average connectivity was *k* = 8 in all beta cell networks. The node degree distributions are presented in Figures 5I,J and were found to be rather heterogeneous and similar for both dynamical components and stimulation levels. Moreover, a weak correlation was identified between the node degrees in networks extracted from fast and slow oscillatory activity. The tendency of better-connected cells harboring more functional connections in both networks was more pronounced under 12 mM than under 8 mM glucose. A comparison of network characteristics showed that the average lengths of functional connections are more than twofold higher in the slow component network layer (Figures 5M,N). This difference was significant and the effect size huge (*p* < 0.001, *d* = 2.82 for 8 mM and *p* = 0.002, *d* = 1.86 for 12 mM glucose). In both glucose concentrations, the fast component network layer exhibited higher clustering levels in comparison to the slow component network (Figures 5O,P; *p* = 0.092, *d* = 0.85 for 8 mM and *p* < 0.001, *d* = 2.20 for 12 mM glucose). Moreover, the network architecture of the fast component was found to be less cohesive (lower relative largest component, Figures 5Q,R), but only under physiological stimulation levels, where the difference compared with the slow component was significant with a large effect size (*p* = 0.053, *d* = 0.98). Under supraphysiological glucose levels the difference was not significant and characterized by a medium effect size (*p* = 0.479, *d* = 0.36). This can be attributed to the fact that high stimulatory conditions evoke high fractions of global waves in the domain of fast activity, which results in very integrated functional connectivity patterns (Markovič et al., 2015; Gosak et al., 2018). Modularity, another network fragmentation metric, also suggested lower levels of integration in the fast component network layer (Figures 5S,T) (*p* = 0.111, *d* = 0.79 for 8 mM and *p* = 0.192, *d* = 0.70 for 12 mM glucose). A higher dispersion of data in this case can probably be attributed to morphological heterogeneity of islets and the resulting inhomogeneous distribution of beta cells in tissue slices.

We wish to suggest that the observed discrepancies in the functional network structures reflect the differences in the spatiotemporal dynamics of intercellular Ca^2+^ waves that coordinate both types of oscillations among different cells. The fast oscillatory activity is being coordinated mostly by gap junction–mediated electrical coupling, resulting in propagating Ca^2+^ waves, which were not always global and encompassed sometimes only a part of the beta cell syncytium. This led to more locally clustered and segregated network structures. While the slow oscillatory component is influenced by gap junctional communication as well, the slow waves were mostly global and occurred over a broader temporal scale. This brought about more long-range connections and more cohesive functional connectivity patterns. However, a systematic analysis and comparison of the nature Ca^2+^ waves coordinating both types of oscillations, as well as the exploration of the underlying mechanisms, is beyond the scope of this article. Finally, it should be noted that in our study we have used variable thresholds to construct functional networks in order to be able to compare the connectivity patterns from different dynamical components, which differ in the degree of correlations between Ca^2+^ signals. We have used an average degree *k* = 8 to mimic realistic beta cell network architectures (Zhang et al., 2008) and to obtain adequately dens networks suitable for analyses. However, within reasonable limits the conclusions do not depend on this rather arbitrary choice of the average degree (see Supplementary Figure 8).

## Discussion

In the present work, we demonstrated that the pancreatic beta cells in mouse tissue slices express a bimodal oscillatory activity of the intracellular Ca^2+^ concentration. Such bimodality of the oscillatory activity was previously described for other stimulus-secretion cascade parameters: the metabolic profile of a beta cell follows the slow oscillatory pattern, and the membrane potential follows the fast temporal pattern (Dean and Matthews, 1968; Santos et al., 1991; Gilon and Henquin, 1992; Liu et al., 1998; Bergsten, 2002; Gilon et al., 2002; Tengholm and Gylfe, 2009; Satin et al., 2015; Skelin Klemen et al., 2017; Bertram et al., 2018). Focusing on the intracellular Ca^2+^ concentration as a surrogate for both metabolic and electrical beta-cell activity, experimental and mathematical modeling studies provided evidence for the Ca^2+^ oscillations resembling the slow metabolic, the fast membrane potential oscillations, or combination of the two. Some studies suggested that our understanding of the two dynamical components might be an experimental artifact. More specifically, it has been suggested that cultivation of isolated islets triggers a phenotype transformation from cells that display fast or compound oscillations to cells with a prevailing slow temporal pattern, likely attributed to the degradation of key membrane proteins by enzymes during the isolation protocol or the conditions used for cultivation of isolated islets (Gilon et al., 1994; Rupnik, 2009). In this study, we employed the acute tissue slice preparation that entirely omits any enzymes during tissue isolation and overnight culture, while preserving both homo- and heterotypic cell-to-cell contacts (Dolenšek et al., 2015). We demonstrated in all preparations and for both stimulatory concentrations of glucose that the beta cells simultaneously display both the slow and the fast Ca^2+^ oscillations. The slow pattern was also detected in basal 6 mM glucose (Figure 3). Together with the glucose-insensitivity of slow oscillations (Figure 2), the above findings present valuable experimental confirmation for similar findings in isolated cells and islets (Gilon et al., 2002; Beauvois et al., 2006; Satin et al., 2015; Bertram et al., 2018; Rorsman and Ashcroft, 2018) and verification for beta-cell models that predict these features (Pedersen et al., 2005; Pedersen, 2009; Merrins et al., 2010; McKenna et al., 2016; Bertram et al., 2018).

In contrast with the slow oscillations, the fast oscillations were modulated by both physiological (8 mM) and supraphysiological (12 mM) stimulation. Both the frequency and the phase duration of the fast component increased with increasing glucose concentration (Figure 2), corroborating earlier studies on isolated islets (Santos et al., 1991; P. Gilon et al., 1993; Antunes et al., 2000; Nunemaker et al., 2006), in acute tissue slices (Markovič et al., 2015; Dolenšek et al., 2020), and of mathematical modeling (Nunemaker et al., 2006; Stamper and Wang, 2019). To study the interplay of the two, we correlated the two frequency domains from the same beta cells (Figure 2). Especially for the physiological concentration (8 mM) and a portion of islets exposed to 12 mM glucose, the slow activity strongly influenced the fast component. More specifically, there was a phase-dependency between both dynamical components, with the highest bursting activity around the maxima and the lowest around the minima of slow oscillations (Figures 2A,C,E,G). The correlation was weaker in other islets in which the fast component seemed less influenced by the slow oscillations (Figures 2B,F,D,H). Increasing stimulation to the supraphysiological levels (12 mM) decreased the overall correlation (Figure 2K), pushing more islets to a more continuous bursting pattern. As in our previous work and studies by others, supraphysiological concentrations were typically used (>11.1 mM), this might explain why the modulation of the fast component was largely overlooked previously. Importantly, our finding that in higher glucose, the fast oscillations also continue during the minima of slow oscillations with almost unaltered frequency implies that increasing glucose increases insulin release through an extension of beta cell activity to otherwise silent or less active periods, but probably at the cost of attenuating the pulsatility of insulin release (Matthews et al., 1983a; Juhl et al., 2000, 2001).

The rhythmogenesis of the oscillatory activity in beta cells has been a controversial topic for decades and has attracted the attention of experimentalists as well from theoretical and computational scientists (Bergsten, 2002; Gilon et al., 2002; Satin et al., 2015; Bertram et al., 2018; Zavala et al., 2019; Grubelnik et al., 2020). For the fast component, it has been proposed that the mechanism involves feedback of Ca^2+^ ions on ion channels. A rise in the intracellular Ca^2+^ concentration activates the calcium-dependent K (K_*Ca*_) channels, causing hyperpolarization and closure of the voltage-dependent Ca^2+^ channels. The latter decreases Ca^2+^ influx triggering a decrease in the Ca^2+^ concentration that ultimately removes the inhibitory drive of the K_*Ca*_ channels, and the cycle can repeat (Nunemaker et al., 2006; Satin et al., 2015). There is no clear consensus on the origin of the beta cell activity’s slow component. These were reported to be in phase with the slow oscillations of the insulin secreted *in vitro* and *in vivo* (Bergsten, 2002; Gilon et al., 2002; Bertram et al., 2018), and are thought to reflect the oscillations in metabolism; therefore, the terms slow and metabolic oscillations are often used interchangeably (Satin et al., 2015). Different studies demonstrated that both the slow and the fast pulses of insulin correlate well with the respective time domains of the Ca^2+^ oscillation dynamics in beta cells. Insulin secretion perfectly matches the slow Ca^2+^ oscillations in isolated islets from mice (Bergsten et al., 1994; Gilon et al., 2002) and humans (Hellman, 2009). Although cultured isolated islets generally exhibit slow oscillations, in a few isolated islets that exhibited frequencies similar to the fast component observed in our preparation (approx. 6 oscillations/minute), the insulin dynamics also perfectly matched these faster Ca^2+^ dynamics (Bergsten, 1995; Barbosa et al., 1998), suggesting that the insulin dynamics can follow Ca^2+^ dynamics even in the faster domain. There is also no clear consensus on whether the oscillatory pattern of the Ca^2+^ drives the slow component (Ca^2+^-driven metabolic oscillations) or *vice versa* (metabolism-driven Ca^2+^ oscillations) (Watts et al., 2014). Experimental data to date provided evidence for either scenario. On the one hand, the oscillations in Ca^2+^ were shown to be a prerequisite for the metabolic component (Gilon et al., 2002; Kennedy et al., 2002; Bertram et al., 2007). On the other hand, perturbing the metabolic oscillations with the a-ketoisocaproic acid (KIC), which enters metabolism at the citric acid cycle, bypassing glycolysis and clamping metabolism levels to a steady-state, affected (albeit inconsistently) the Ca^2+^ oscillations (Bertram et al., 2018). While some studies reported KIC-induced slow oscillations (Martin et al., 1995), others failed to reproduce the KIC effect (Lenzen et al., 2000; Dahlgren et al., 2005). In this study, we found a clear glucose-dependence of fast Ca^2+^ oscillations with respect to time required for their initiation, their frequency, and the active time. In contrast, the slow oscillations did not show any glucose-dependence, at least in the investigated range of concentrations, and they also existed in the absence of the fast component (in 6 mM glucose). Moreover, the frequency of the fast component depended on the phase of the slow component. These facts, taken together, imply that the mechanism driving the slow activity of intracellular Ca^2+^ concentration is distinct from the one responsible for the fast component, that the slow component influences the fast, but that the presence of fast oscillations and their characteristics do not influence the slow oscillations. More specifically, the slow oscillations cause shifts in the frequency of the fast oscillations, but the average value of these fast oscillations is set by a glucose-dependent mechanism, distinct from the one responsible for slow oscillations. This corroborates the recent developments in computational models of beta cell activity, suggesting that the slow oscillations may originate from intrinsic mechanisms, in addition to Ca^2+^ effects on the enzymes involved in beta cell metabolism (McKenna et al., 2016; Bertram et al., 2018; Fazli et al., 2020). We wish to point out that our method of measuring changes in Ca^2+^ does not enable assessments of absolute changes in amplitudes and thus we cannot completely exclude the possibility that the amplitude of the slow oscillations may be glucose-dependent. We also never observed fast oscillations without any underlying slow oscillations, but this does not mean that such a pattern of activity does not exist.

Proper pulsatile secretory responses require the beta cells to work in synchrony, which is ensured by gap junctions, other modes of intercellular communication, and by paracrine signals (Bavamian et al., 2007; Benninger et al., 2011, 2014; Bosco et al., 2011; Head et al., 2012; Skelin Klemen et al., 2017; Benninger and Hodson, 2018; Almaça et al., 2020; Lammert and Thorn, 2020). The former is the main synchronizing mechanism of the fast oscillatory domain by facilitating the propagation of depolarization and Ca^2+^ waves across the islets. Collective behavior of fast oscillations is receiving much more attention from the scientific community, especially because inherent beta cell heterogeneity and the existence of subpopulations lead to complex spatio-temporal activity patterns, characterized by heterogeneous and non-stationary intercellular waves, which are also accessible to experimental and modeling approaches (Hraha et al., 2014; Cappon and Pedersen, 2016; Gosak et al., 2017; Westacott et al., 2017b; Šterk et al., 2021). These waves are typically initiated from subregions with elevated excitability (Benninger et al., 2014) and with increasing glucose concentration, they become more global (Stožer et al., 2019), which results in more integrated functional network structures, as we have also observed in the present study (Figure 5). In contrast, the characteristics of slow collective activity and the underlying mechanisms are much less known. Our results clearly indicate that the slow oscillations are not only rather well aligned across the islets, but also that nearby cells are better synchronized than remote ones (see Figures 3, 4). More specifically, our results imply two conclusions: first, in the slow oscillations domain, not all cells in the islet are simultaneously in the same phases. Second, there must be some synchronizing mechanism that promotes the coordination of slow oscillations among neighboring cells. This might be the diffusion of glucose-6-phosphate or some other metabolic intermediate (Tsaneva-Atanasova et al., 2006; Loppini et al., 2015) or the indirect influence of the feedback of well-aligned fast component oscillations. Theoretically, it has been suggested that gap junction-mediated electrical coupling, diffusion of glycolytic intermediates, or a combination of both can contribute to the synchronization of slow oscillations (Pedersen et al., 2005). However, to what extent different means of intercellular communication shape the complex spatio-temporal activity in islets, remains to be elucidated. Moreover, we argue that the first point about the different phases of the slow component might refer to the multifaceted heterogeneity of beta cells, which results in the existence of subpopulations with similar cellular signaling characteristics (Dwulet et al., 2019; Stožer et al., 2019; Da Silva Xavier and Rutter, 2020; Rutter et al., 2020). This would also explain the relatively high abundance of long-range connections in the network extracted from the dynamical slow component (see Figures 4E,G), which link different subpopulations with similar metabolic profiles. In contrast, in the fast component network, connections interconnect particularly cells within the same subgroup, whereas long-range connections are manifested mostly only by specific hub cells (Markovič et al., 2015; Johnston et al., 2016; Gosak et al., 2018). From a functional point of view, the slow component seems to set the pace for all cells within an islet and ensure that cells in different regions are all active during the same periods, whereas the fast component probably fine-tunes the number of cells that are recruited during an active period, as well as their level of activity. Nevertheless, further studies will be necessary for elucidating the precise mechanisms that govern the intercellular synchronicity of different dynamical components in beta cells, for instance by systematically comparing the characteristics of intercellular Ca^2+^ waves synchronizing the fast and slow oscillations. We will also have to define the roles that beta cells play across networks extracted from different temporal domains, find out whether these roles are stable and dependent on long-term processes, such as differentiation, or more flexible and dependent on local cues, and explore how they contribute to normal and pathological endocrine function.

To conclude, insulin secretion, as well as other metabolic and hormonal rhythms, are ubiquitous and vital for maintaining normal physiological functions. These rhythms result from the interplay between several feedback systems and occur at multiple timescales and levels of organization (Corkey and Shirihai, 2012; Bertram et al., 2018). The recently emerging fields of network physiology and network medicine show great potential to address such issues and to provide new insights into how global behavior at the organism level can arise out of micro-mechanisms on the cellular and tissue level (Bashan et al., 2012; Ivanov et al., 2016). Metabolic systems make excellent candidates for being studied by these novel interdisciplinary approaches (Zavala et al., 2019; Corkey and Deeney, 2020; Martinez et al., 2020). Understanding how the multimodal activity of beta cells acts in synchrony and integrates to the organ level, how heterologous interactions with other islet cells affect the pancreatic output, how the complementary action of other hormones contributes to the dynamic crosstalk between metabolic organs, and how all these pathways are impaired in diabetes, are some of the main questions in islet and in specific metabolic diseases research (Rorsman and Ashcroft, 2018; Rutter et al., 2020). We firmly believe that addressing these issues will require new perspectives and integrative frameworks based on tools developed in the field of network science and computational physiology, which will support and complement experimental endeavors.

## Data Availability Statement

The raw data supporting the conclusions of this article will be made available by the authors, without undue reservation.

## Ethics Statement

The animal study was reviewed and approved by Administration of the Republic of Slovenia for Food Safety, Veterinary Sector and Plant Protection (permit number: 34401-35-2018/2).

## Author Contributions

MSK, JD, and AS performed the experiments. JZ, RM, and MG developed the software for the analysis. JZ analyzed the data. JZ and RM prepared the figures. MSK, JD, AS, and MG wrote the manuscript. MM and AS provided resources. MG supervised the study. All authors conceived the idea, designed the study, and reviewed and approved the manuscript.

## Conflict of Interest

The authors declare that the research was conducted in the absence of any commercial or financial relationships that could be construed as a potential conflict of interest.

## Supplementary Material

Supplementary Video 1 Animation of binarized fast spatiotemporal Ca^2+^ activity in a representative islet under stimulation with 8 mM glucose and the corresponding raster plot of all binarized traces in the islet.

Supplementary Video 2 Animation of binarized fast spatiotemporal Ca^2+^ activity in a representative islet under stimulation with 12 mM glucose and the corresponding raster plot of all binarized traces in the islet.

Supplementary Video 3 Animation of slow spatiotemporal Ca^2+^ activity in a representative islet under stimulation with 8 mM glucose and the corresponding color-coded plot of phases of the slow oscillatory components for all cells in the islet.

Supplementary Video 4 Animation of slow spatiotemporal Ca^2+^ activity in a representative islet under stimulation with 12 mM glucose and the corresponding color-coded plot of phases of the slow oscillatory components for all cells in the islet.
