# Supplementary material for: Assessing Different Temporal Scales of Calcium Dynamics in Networks of Beta Cell Populations
Source: Front Physiol. 2021 Mar 23;12:612233. doi: 10.3389/fphys.2021.612233 (PMC8021717; doi:10.3389/fphys.2021.612233)
Supplement: Supplementary file 5 [file Data_Sheet_1.PDF]

Supplementary Material

1 Intra- and inter-islet variability

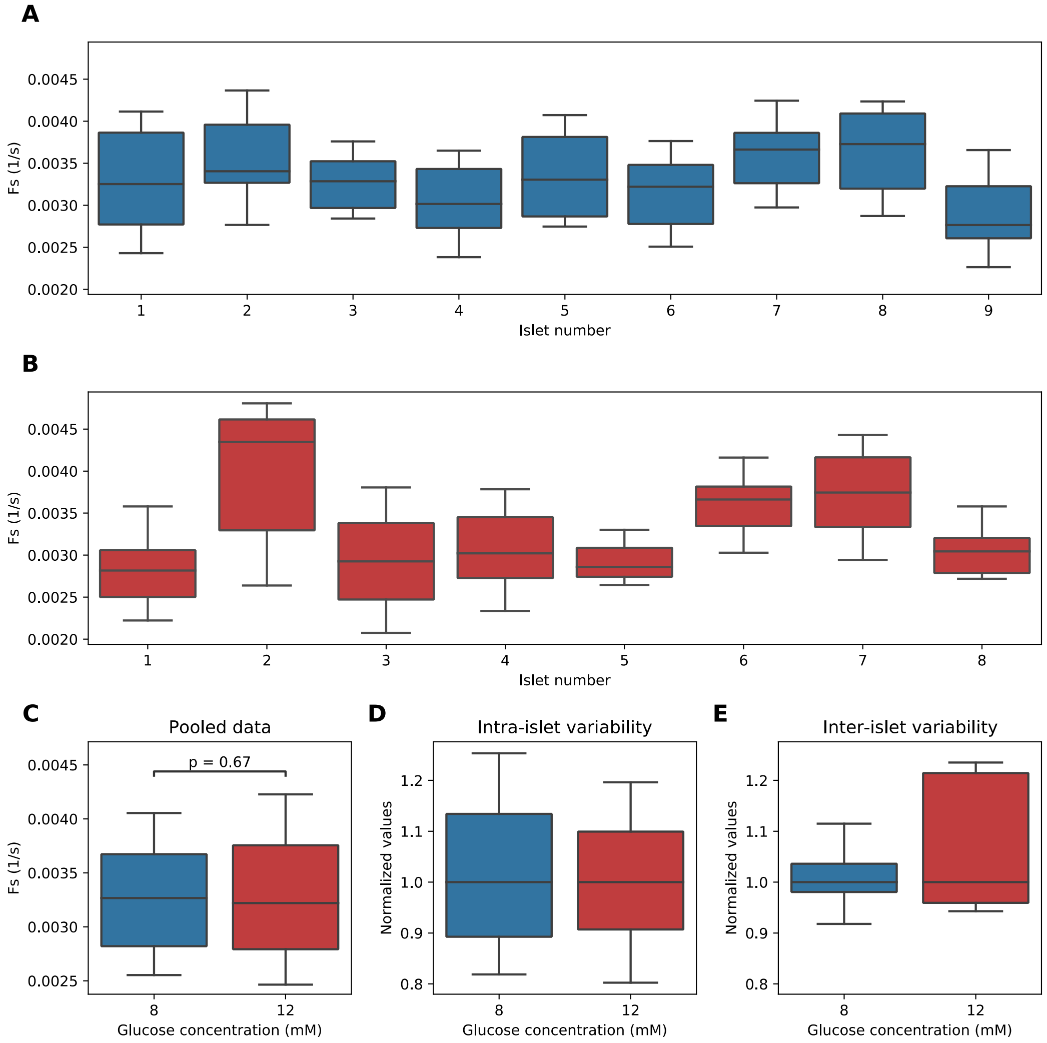

Supplementary Figure 1. Frequencies of the slow oscillatory intracellular Ca^{2+} activity in individual pancreatic beta cells. Distributions of frequencies of the slow oscillatory component in individual islets stimulated with 8 mM (A) and 12 mM glucose (B). The box-plots in panel (C) represent the pooled data of all cells from all islets for a given glucose concentration. Cohen's *d* value is 0.02. Panels (D) and (E) feature the relative dispersion of values within individual islets (intra-islet variability, D) and between islets (inter-islet variability, E). In panel (D) the normalized values were obtained by dividing the frequencies of all cells in each islet by the corresponding median frequency in the given islet. Normalized values in panel (E) were obtained by dividing the median frequencies of each islet by the respective median frequency of slow oscillations from all islets for each glucose concentration. In the diagrams, boxes determinate the interval within 25th and 75th percentiles and whiskers within the 10th and 90th percentiles, whereas the lines indicate the median value. The number of beta cells in different islets was in 8 mM glucose: 92 (1), 108 (2), 103 (3), 107 (4), 134 (5), 65 (6), 123 (7), 19 (8), 178 (9), and in 12 mM glucose: 75 (1), 74 (2), 77 (3), 160 (4), 73 (5), 103 (6), 121 (7), 69 (8).

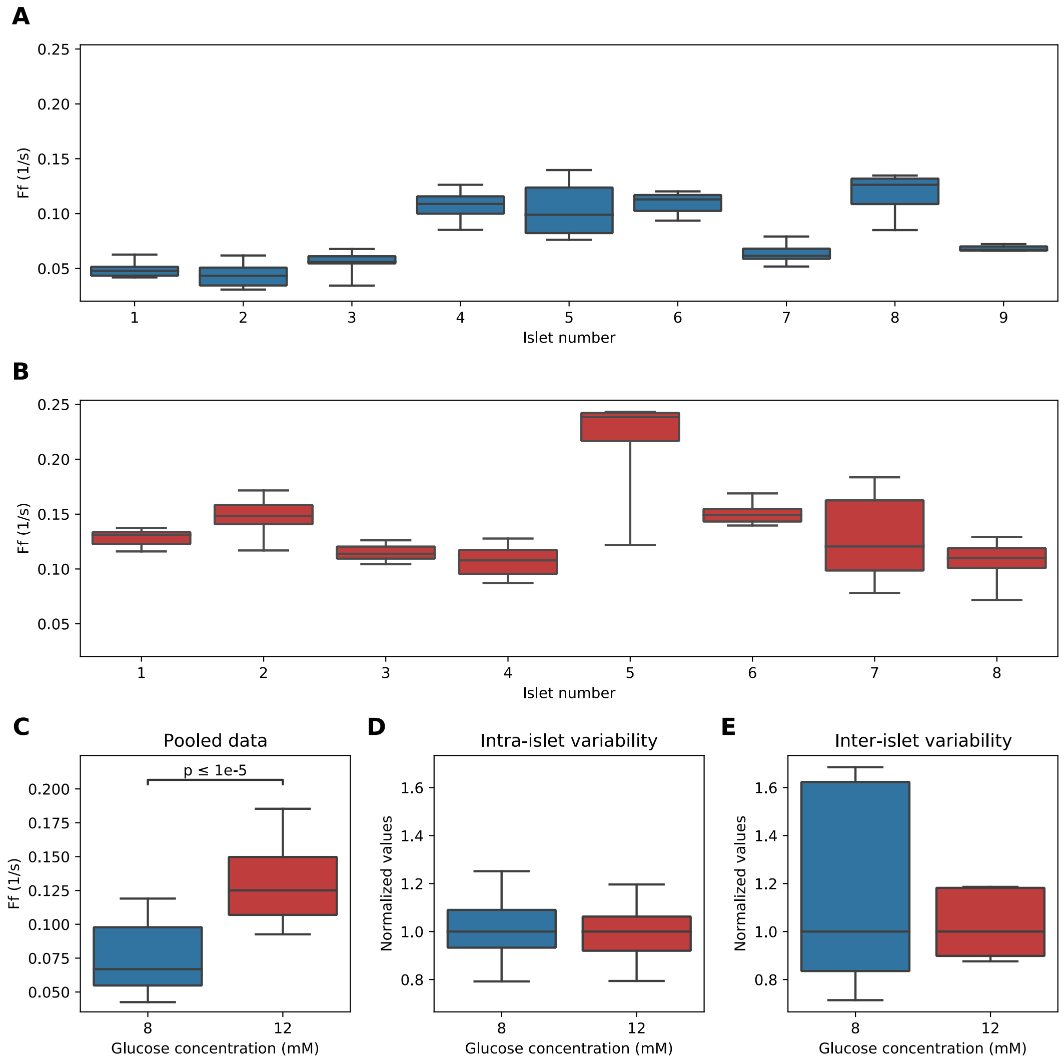

Supplementary Figure 2. Frequencies of the fast oscillatory intracellular Ca^{2+} activity in individual pancreatic beta cells. Distributions of frequencies of the fast oscillatory component in individual islets stimulated with 8 mM (A) and 12 mM glucose (B). The box-plots in panel (C) represent the pooled data of all cells from all islets for a given glucose concentration. Cohen's *d* value is 1.66. Panels (D) and (E) feature the relative dispersion of values within individual islets (intra-islet variability, D) and between islets (inter-islet variability, E). In panel (D) the normalized values were obtained by dividing the frequencies of all cells in each islet by the corresponding median frequency in the given islet. Normalized values in panel (E) were obtained by dividing the median frequencies of each islet by the respective median frequency of fast oscillations from all islets for each glucose concentration. Box charts are defined the same as in Supplementary Figure 1.

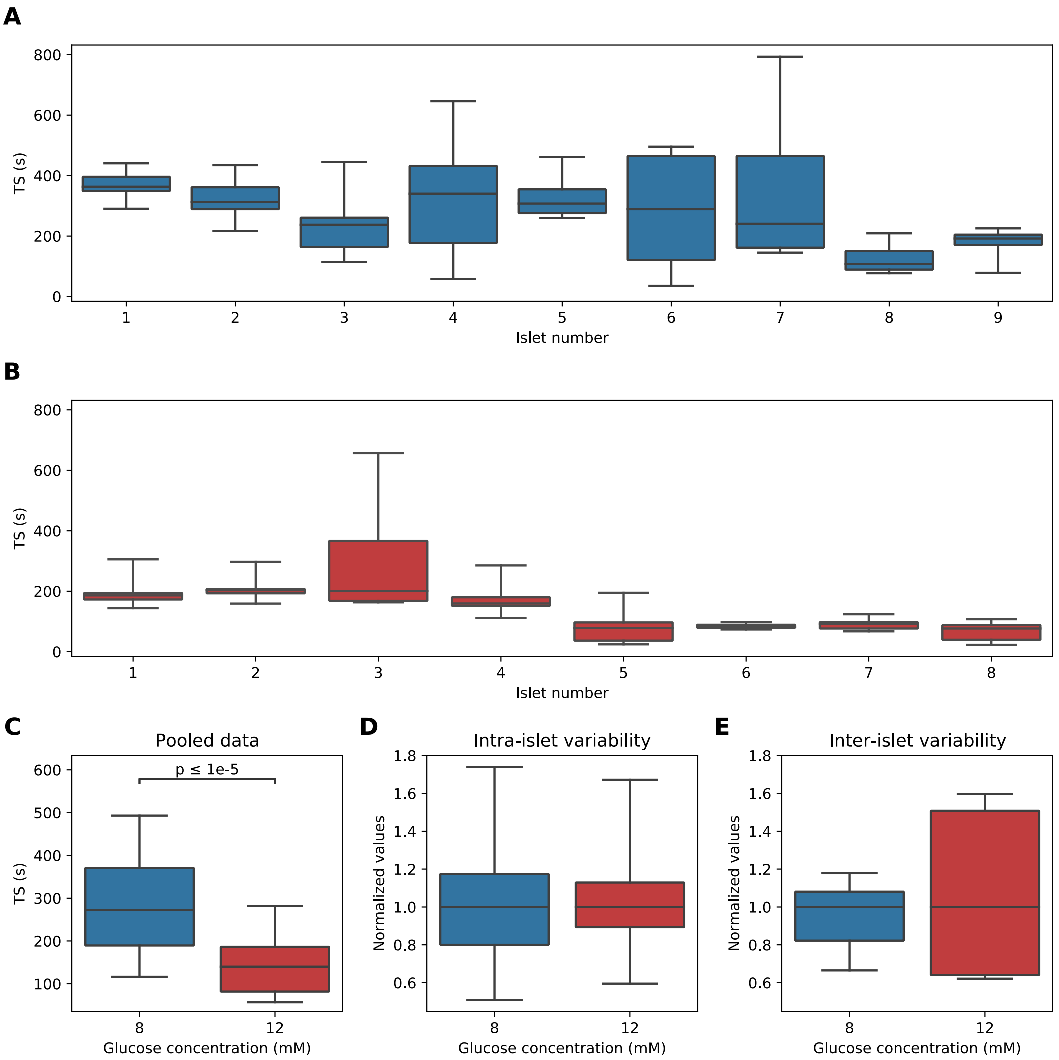

Supplementary Figure 3. Times required for the cells to respond to stimulation (TS) in individual pancreatic beta cells. Distributions of TS in individual islets stimulated with 8 mM (A) and 12 mM glucose (B). The box-plots in panel (C) represent the pooled data of all cells from all islets for a given glucose concentration. Cohen's *d* value is 0.69. Panels (D) and (E) feature the relative dispersion of values within individual islets (intra-islet variability, D) and between islets (inter-islet variability, E). In panel (D) the normalized values were obtained by dividing the TS of all cells in each islet by the corresponding median TS in the given islet. Normalized values in panel (E) were obtained by dividing the median TS values of each islet by the respective median TS from all islets for each glucose concentration. Box charts are defined the same as in Supplementary Figure 1.

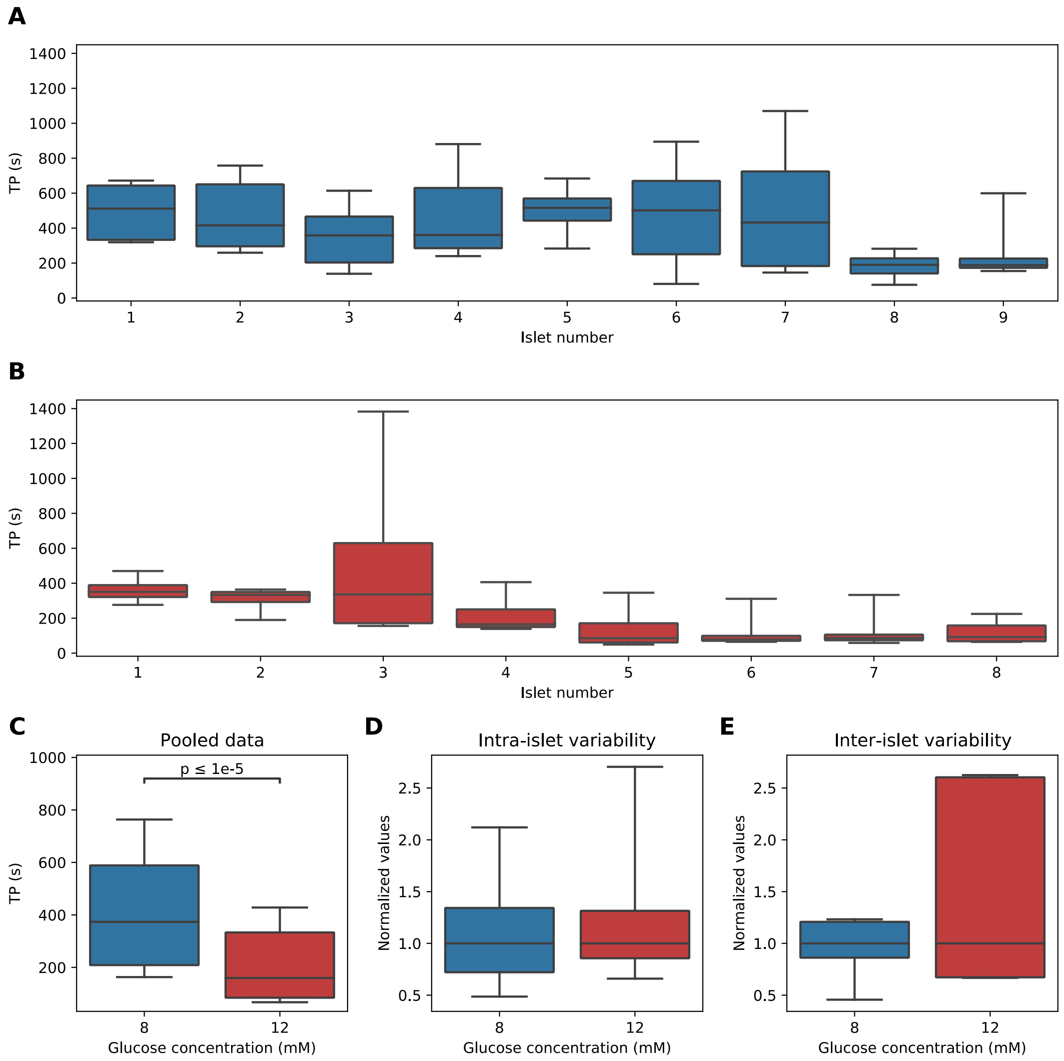

Supplementary Figure 4. Times required for the cells to reach the phase of sustained activity, i.e. the plateau phase (TP) in individual pancreatic beta cells. Distributions of TP in individual islets stimulated with 8 mM (A) and 12 mM glucose (B). The box-plots in panel (C) represent the pooled data of all cells from all islets for a given glucose concentration. Cohen's *d* value is 0.71. Panels (D) and (E) feature the relative dispersion of values within individual islets (intra-islet variability, D) and between islets (inter-islet variability, E). In panel (D) the normalized values were obtained by dividing the TP of all cells in each islet by the corresponding median TP in the given islet. Normalized values in panel (E) were obtained by dividing the median TP values of each islet by the respective median TP from all islets for each glucose concentration. Box charts are defined the same as in Supplementary Figure 1.

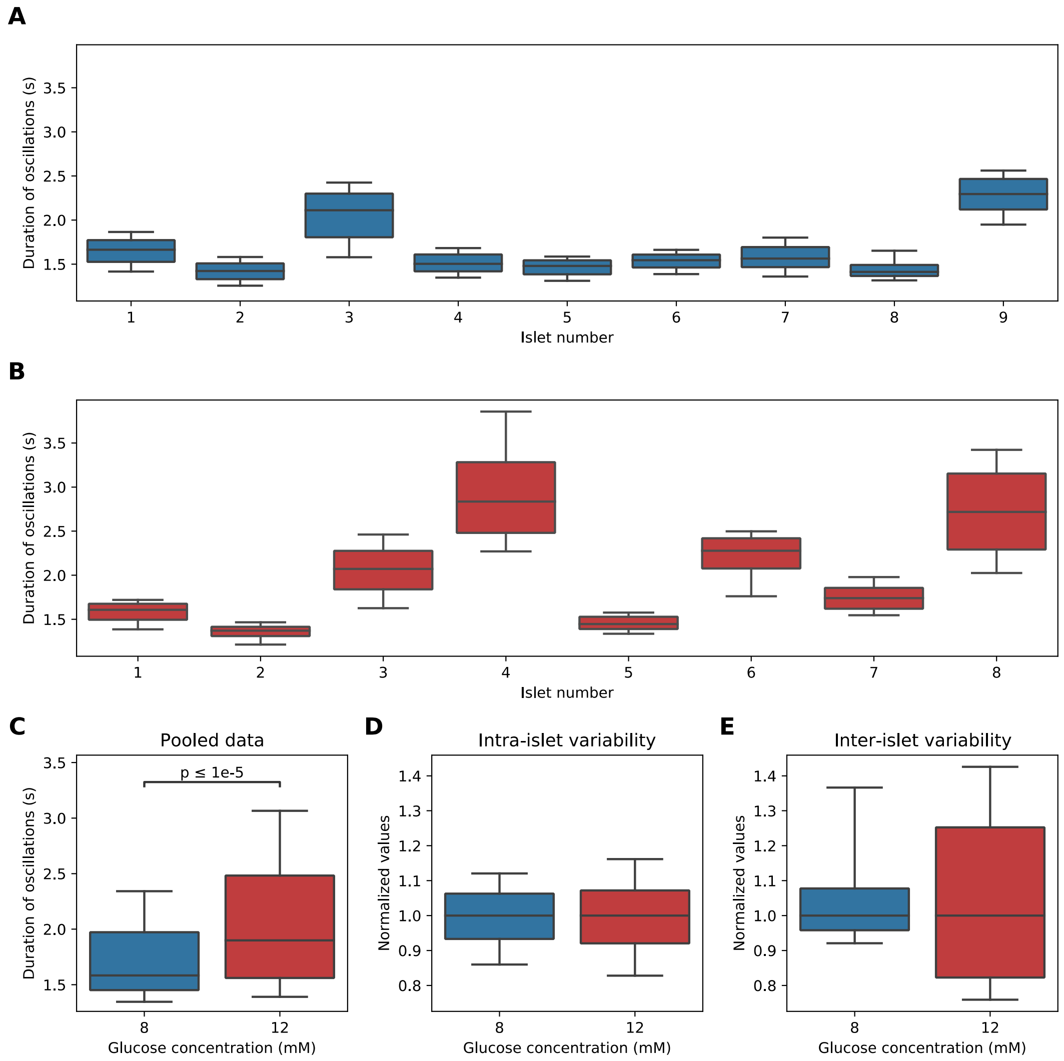

Supplementary Figure 5. Durations of individual oscillations in individual pancreatic beta cells. Distributions of durations of in individual islets stimulated with 8 mM (A) and 12 mM glucose (B). The box-plots in panel (C) represent the pooled data of all cells from all islets for a given glucose concentration. Cohen's *d* value is 0.70. Panels (D) and (E) feature the relative dispersion of values within individual islets (intra-islet variability, D) and between islets (inter-islet variability, E). In panel (D) the normalized values were obtained by dividing the durations of all cells in each islet by the corresponding median duration in the given islet. Normalized values in panel (E) were obtained by dividing the median durations of each islet by the respective median duration from all islets for each glucose concentration. Box charts are defined the same as in Supplementary Figure 1.

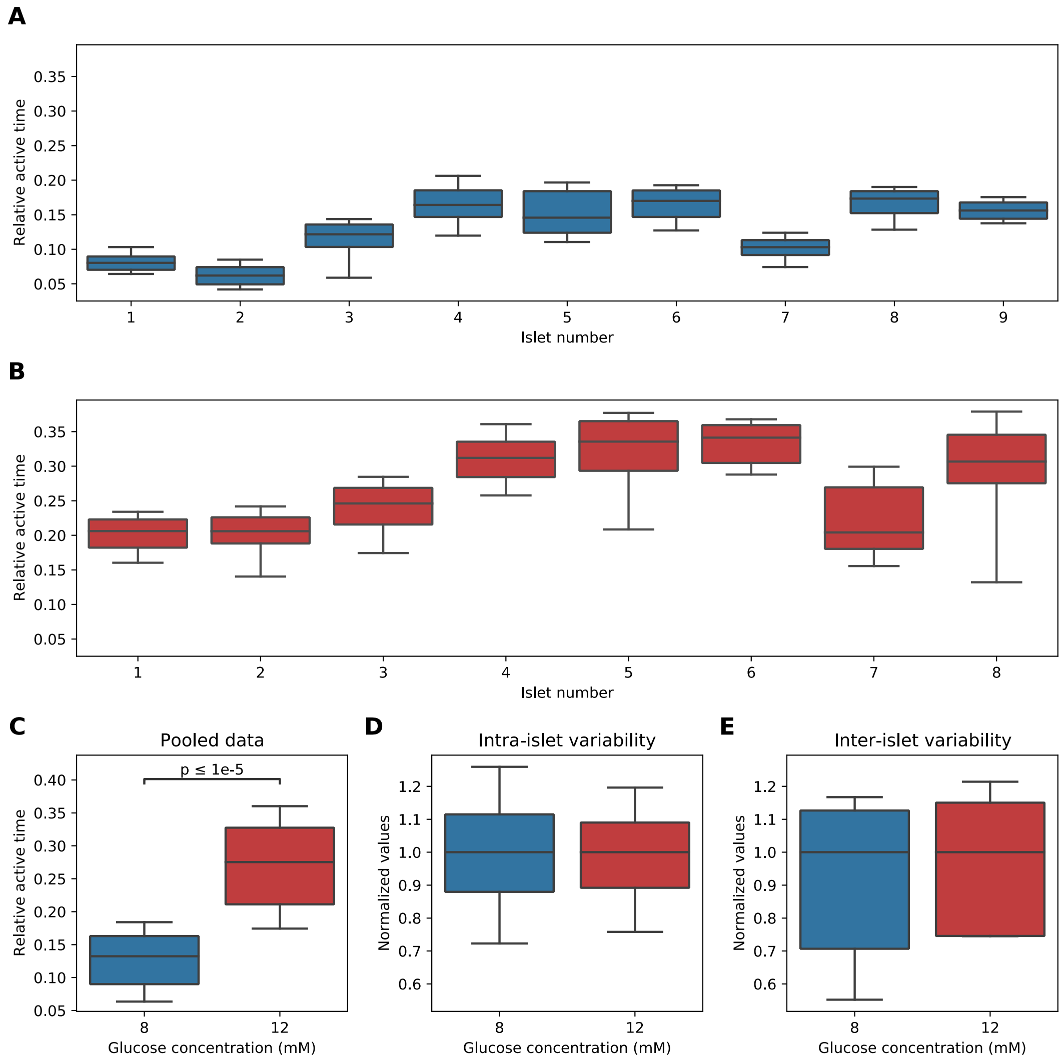

Supplementary Figure 6. Relative active times (ATs) in individual pancreatic beta cells. Distributions of ATs in individual islets stimulated with 8 mM (A) and 12 mM glucose (B). The box-plots in panel (C) represent the pooled data of all cells from all islets for a given glucose concentration. Cohen's *d* value is 2.35. Panels (D) and (E) feature the relative dispersion of values within individual islets (intra-islet variability, D) and between islets (inter-islet variability, E). In panel (D) the normalized values were obtained by dividing the ATs of all cells in each islet by the corresponding median AT in the given islet. Normalized values in panel (E) were obtained by dividing the median ATs of each islet by the respective median AT from all islets for each glucose concentration. Box charts are defined the same as in Supplementary Figure 1.

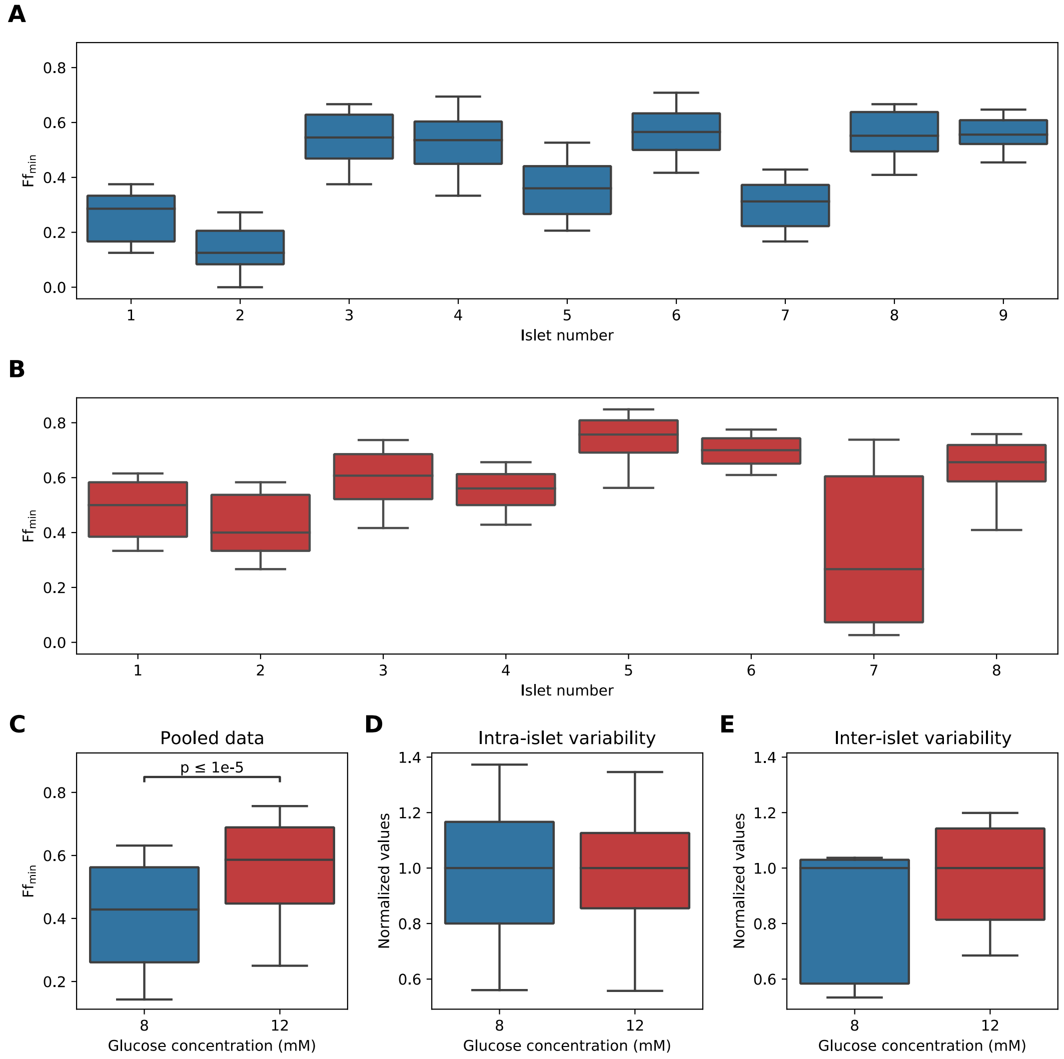

Supplementary Figure 7. The lowest values of slow-phase-dependent relative density of fast oscillations (lowest relative densities) in individual pancreatic beta cells. Distributions of lowerst relative frequencies in individual islets stimulated with 8 mM (A) and 12 mM glucose (B). The box-plots in panel (C) represent the pooled data of all cells from all islets for a given glucose concentration. Cohen's *d* value is 0.69. Panels (D) and (E) feature the relative dispersion of values within individual islets (intra-islet variability, D) and between islets (inter-islet variability, E). In panel (D) the normalized values were obtained by dividing the relative lowerst frequencies of all cells in each islet by the corresponding median lowest relative frequency in the given islet. Normalized values in panel (E) were obtained by dividing the median lowerst relative frequencies of each islet by the respective median lowest relative frequency Ff_{min} from all islets for each glucose concentration. Box charts are defined the same as in Supplementary Figure 1.

2 Robustness of the functional beta cell network parameters with respect to changes in the average node degree

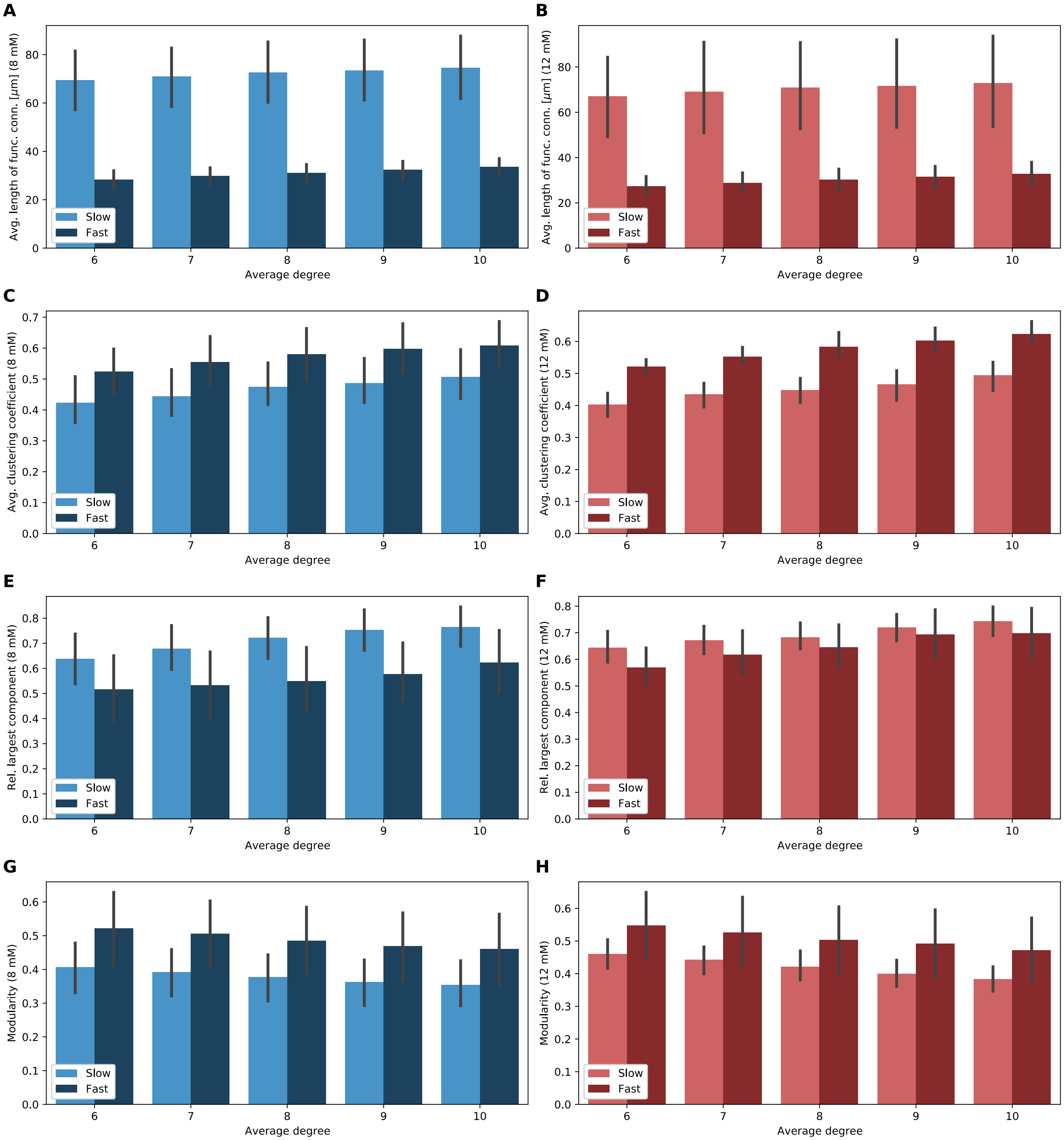

Supplementary Figure 8. Robustness of the functional beta cell network parameters with respect to changes in the average node degree. Different network metrics from functional connectivity maps with different average node degrees, which were extracted from the intercellular Ca^{2+} activity at low (A, C, E, G) and at high (B, D, F, H) glucose concentration. Light blue and light red bars correspond to the slow component, whereas the dark blue and dark red bars correspond to the fast oscillatory component.
